# A U-shaped association of tracheostomy timing with all-cause mortality in mechanically ventilated patients admitted to the intensive care unit: A retrospective cohort study

**DOI:** 10.3389/fmed.2022.1068569

**Published:** 2022-12-14

**Authors:** Jing-Ran Chen, Hao-Ran Gao, Yan-Lin Yang, Yan Wang, Yi-Min Zhou, Guang-Qiang Chen, Hong-Liang Li, Linlin Zhang, Jian-Xin Zhou

**Affiliations:** ^1^Department of Critical Care Medicine, Beijing Tiantan Hospital, Capital Medical University, Beijing, China; ^2^Department of Critical Care Medicine, Beijing Ditan Hospital, Capital Medical University, Beijing, China; ^3^Department of Critical Care Medicine, Beijing Shijitan Hospital, Capital Medical University, Beijing, China

**Keywords:** intensive care unit, tracheostomy, mechanical ventilation, clinical outcomes, extubation

## Abstract

**Objectives:**

To evaluate the association of tracheostomy timing with all-cause mortality in patients with mechanical ventilation (MV).

**Method:**

It’s a retrospective cohort study. Adult patients undergoing invasive MV who received tracheostomy during the same hospitalization based on the Medical Information Mart for Intensive Care-III (MIMIC-III) database, were selected. The primary outcome was the relationship between tracheostomy timing and 90-day all-cause mortality. A restricted cubic spline was used to analyze the potential non-linear correlation between tracheostomy timing and 90-day all-cause mortality. The secondary outcomes included free days of MV, incidence of ventilator-associated pneumonia (VAP), free days of analgesia/sedation in the intensive care unit (ICU), length of stay (LOS) in the ICU, LOS in hospital, in-ICU mortality, and 30-day all-cause mortality.

**Results:**

A total of 1,209 patients were included in this study, of these, 163 (13.5%) patients underwent tracheostomy within 4 days after intubation, while 647 (53.5%) patients underwent tracheostomy more than 11 days after intubation. The tracheotomy timing showed a U-shaped relationship with all-cause mortality, patients who underwent tracheostomy between 5 and 10 days had the lowest 90-day mortality rate compared with patients who underwent tracheostomy within 4 days and after 11 days [84 (21.1%) vs. 40 (24.5%) and 206 (31.8%), *P* < 0.001].

**Conclusion:**

The tracheotomy timing showed a U-shaped relationship with all-cause mortality, and the risk of mortality was lowest on day 8, but a causal relationship has not been demonstrated.

## Introduction

Tracheostomy is one of the most used procedures in mechanically ventilated patients during the intensive care unit (ICU) stay (1, 2). The expected benefits of tracheostomy include enhanced comfort, improved pulmonary drainage, and decreased sedation depth. However, tracheostomy may also raise the risk of complications such as bleeding, incision infection, and tracheal stenosis. The optimal timing of tracheostomy remains unclear because uncertainty exists in the balance between the benefits and risks of tracheostomy (3, 4). Given the influence of tracheostomy timing on clinical outcomes, including the incidence of ventilator-associated pneumonia (VAP), duration of mechanical ventilation, length of stay (LOS) in the ICU and hospital, and mortality, observational studies with large sample sizes (5–12) and recent systematic reviews of randomized controlled trials (13–18) have yielded conflicting results, which might be due mainly to the diversity in patient populations and definitions of early versus late tracheostomy among different investigations.

Mechanically ventilated patients admitted to the ICU represent a heterogeneous group of illnesses. Certain categories of critically ill patients, such as acute brain injury, might benefit from early tracheostomy (19). Regarding the timing of tracheostomy, the definition of “early” in previous studies was also different, ranging from 3 to 21 days after mechanical ventilation (5–18). The timing of tracheostomy is a continuous variable. Binary classification to “early” and “late” might be unsuitable for exploring the effect of tracheostomy timing on clinical outcomes. Therefore, we constructed a non-linear regression model with adjustment of confounders between tracheostomy timing and 90-day all-cause mortality in different classifications of critically ill patients receiving mechanical ventilation.

## Materials and methods

### Data source

We performed a retrospective observational study using the Medical Information Mart for Intensive Care-III (MIMIC-III) database, containing de-identified datasets of 53,423 adult patients admitted to the ICUs in the Beth Israel Deaconess Medical Center in Boston (BIDMC) between 2001 and 2012 (20). MIMIC-III is an open-source database derived from two different information systems, the Philips CareVue Clinical Information System (hereinafter referred to as CareVue) and the iMDsoft MetaVision ICU (hereinafter referred to as MetaVision). CareVue and MetaVision recorded patients admitted to the hospital from 2001 to 2008 and after 2008, respectively. Data in MIMIC-III were downloaded from three sources, including archives from critical care information systems and hospital electronic health record databases for in-hospital data and the Social Security Administration Death Master File, which contained the follow-up time and out-of-hospital mortality. The establishment of this database was approved by the Massachusetts Institute of Technology (Cambridge, MA, USA) and Beth Israel Deaconess Medical Center (Boston, MA, USA), and consent was obtained for the original data collection. Therefore, the ethical approval statement and the need for informed consent were waived for this research by the Institutional Review Board of Beijing Tiantan hospital. The study was designed and conducted in accordance with relevant guidelines and regulations (Declaration of Helsinki).

### Study cohort

Data were extracted from all adult patients who were documented with mechanical ventilation and tracheostomy during the same hospitalization. For patients with multiple admissions to the ICU and those with multiple tracheostomy procedures, we collected only the first admission and the first tracheotomy data. Our inclusion criteria were: (1) adult patient aged 18 years or older; (2) accepted mechanical ventilation and tracheostomy in the same hospitalization; and (3) the time of tracheostomy could be obtained. Our exclusion criteria were: (1) the intubation time before tracheostomy was not recorded; (2) successfully extubated before tracheostomy was performed; and (3) patients who were not first hospitalized. To obtain our exposure of interest, which is the timing of tracheostomy, the following search strategy was developed. And all data were extracted using the Structured Query Language (SQL).

**FIGURE 1:**
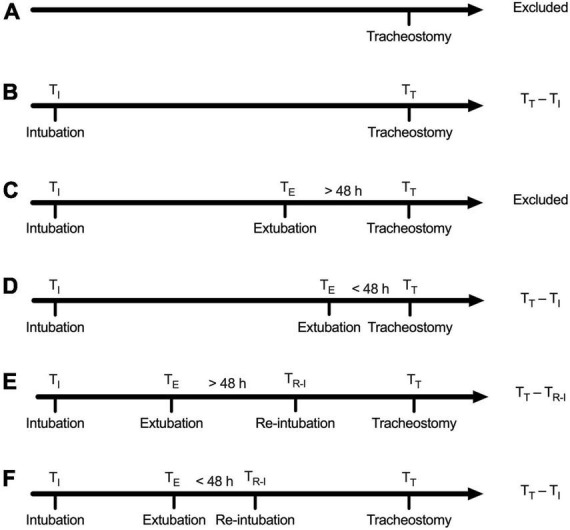
Definition of tracheostomy timing. **(A)** Patients with no record of endotracheal intubation regardless of the record of extubation were excluded. **(B)** Cases with only one record of intubation and without the record of extubation: Tracheostomy timing was calculated as the time interval between intubation and tracheostomy. **(C)** Tracheostomy was performed beyond 48 h after extubation: Defined as successful extubation and excluded. **(D)** Tracheostomy was performed within 48 h after extubation, defined as extubation failure, and tracheostomy timing was calculated as the time interval between tracheostomy and intubation prior to failed extubation. **(E)** Reintubation was performed beyond 48 h after extubation, defined as successful extubation, and tracheostomy timing was calculated as the time interval between reintubation and tracheostomy. **(F)** Reintubation was performed within 48 h after extubation, defined as extubation failure, and tracheostomy timing was calculated as the time interval between the first intubation and the tracheostomy.

The MIMIC-III database is derived from the CareVue and MetaVision clinical information systems. Tracheostomy is documented as an operational event in the MetaVision system. The type of tracheostomy is also recorded. However, tracheostomy is not recorded as an operational event in the CareVue system. We retrieved the CareVue system using keywords including tracheostomy cuff, tracheostomy type, and tracheostomy care. The first record relating to tracheostomy was defined as the time of tracheostomy. The timing of tracheostomy was defined as the time interval between endotracheal intubation and tracheostomy. Six possible situations that existed in the collected datasets were shown in Figure 1 based on the criteria in the guideline (21).

### Outcomes

The primary clinical outcome was 90-day all-cause mortality. Cases recorded in the CareVue system were followed for at least 4 years, whereas those recorded in the MetaVision system were followed for at least 90 days.

The secondary outcomes included duration of mechanical ventilation, free days of MV, incidence of ventilator-associated pneumonia (VAP), free days of analgesia/sedation in the intensive care unit (ICU), length of stay (LOS) in the ICU, LOS in hospital, in-ICU mortality, and 30-day all-cause mortality.

### Possible confounding factors

We collected possibly confounding factors that may affect 90-day mortality, including demographics, the severity of illness, and admission type. The severity of illness was represented by the Simplified Acute Physiology Score (SAPS) II (22) and Sequential Organ Failure Assessment (SOFA) (23) in the first 24 h of ICU admission. The Elixhauser Comorbidity Index (ECI) was used to assess the burden of comorbidities (24, 25) according to an algorithm provided by the authors of the MIMIC-III database, we quote the one named “elixhauser_score_quan.sql” (26). And Glasgow Coma Scale (GCS) in the first 24 h of ICU admission is also extracted. In order to further study the effect of tracheostomy timing on mortality in different comorbidities and diseases, the following four types of comorbidities were mainly concerned: neurological disease, tumor, respiratory disease, and cardiac disease. Neurological disease includes paralysis and other neurological disorders; tumor includes metastatic cancer, lymphoma, and solid tumor without metastasis; respiratory disease includes chronic pulmonary disease; cardiac disease includes congestive heart failure, cardiac arrhythmias, and valvular disease. Considering that different levels of consciousness may also influence the timing of tracheostomy, we also performed a subgroup analysis of patients according to different GCS scores on admission. We also identified three particular diseases: acute respiratory failure, brain injury and sepsis. Acute respiratory failure was identified from the International Classification of Diseases, Ninth Revision (ICD-9). Brain injury including traumatic brain injury (TBI), subarachnoid hemorrhage (SAH), other intracerebral hemorrhage (ICH), intracranial space occupying lesion, and intracranial infections were identified from ICD-9 as well. Patients with sepsis were identified based on established definitions (27). Based on the small fraction of missing data, casewise deletion was used to deal with it.

### Statistical analysis

We aimed primarily to determine the dose-response relationship between tracheostomy timing and all-cause mortality in ICU patients. Univariate and multivariate COX proportional hazard regression analyses with stepwise elimination (variables presenting *P* < 0.05 and were included in multivariate regression analyses) were used to identify the association between tracheostomy timing and 90-day all-cause mortality. Then, we used a restricted cubic spline Cox regression model to further analyze the non-linear association between tracheostomy timing and the risk of 90-day all-cause mortality. The restricted cubic splines we used had five knots at the 5th, 27.5th, 50th, 72.5th, and 95th centiles to flexibly model the association of tracheostomy timing with mortality (28, 29), and the reference point was the median tracheostomy timing. Then, we did a subgroup analysis tended to find the difference in tracheostomy timing in different classifications of critically ill patients receiving mechanical ventilation.

For continuous variables including age, data were reported as the median and quartile. Ranked data, such as SOFA score, SAPS II score, ECI score, and GCS score are reported as the median quartile. Two-tailed *T*-test or Mann–Whitney *U* test was used to compare differences between the two groups. Classified data such as tracheotomy method, type of admission, and type of ICU admission for the first time were described in terms of rate or percentage and compared by the chi-square test or Fisher’s exact test. *P*-value was adjusted with Bonferroni’s method in multiple comparisons. The R software program, version 4.0.5,^1^ and the SPSS program, version 26.0 (IBM Corporation, Armonk, NY, USA) were used for statistical analyses. A two-tailed *P*-value of 0.05 was considered significant for all analyses.

## Results

### Patient characteristics

**FIGURE 2:**
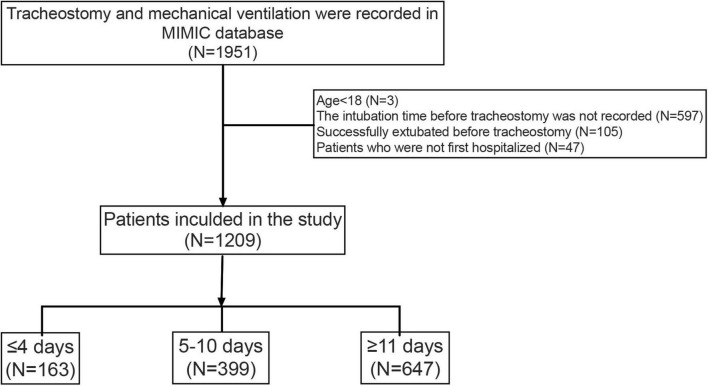
Cohort selection.

A total of 1,951 hospitalizations with tracheostomy and MV records were retrieved. Based on our exclusion criteria, 1,209 patients were selected for the study. Of these, 163 (13.5%) patients underwent tracheostomy within 4 days after intubation, while 647 (53.5%) patients underwent tracheostomy more than 11 days after intubation. Figure 2 shows details of cohort selection. Table 1 describes the baseline and clinical characteristics of the cohort.

**TABLE 1:** Baseline in different tracheostomy timing.

	Tracheostomy timing			
	
Variables	All patients	≤4 days	5–10 days	≥11 days
Tracheostomy timing, median (IQR)	11 (7–16)	3 (2–4)	8 (6–9)	16 (13–20)
**First care unit, no. (%)**				
TSICU/SICU	599 (49.5)	83 (50.9)	250 (62.7)	266 (41.1)
MICU	252 (20.8)	34 (20.9)	64 (16.0)	154 (23.8)
CCU/CSRU	358 (29.6)	46 (28.2)	85 (21.3)	227 (35.1)
**Admission type, no. (%)**				
Non-elective	1108 (91.6)	150 (92.0)	374 (93.7)	584 (90.3)
Elective	101 (8.3)	13 (8.0)	25 (6.3)	63 (9.7)
Gender (no.% Male)	700 (57.9)	99 (60.7)	234 (58.6)	367 (56.7)
Age, median (IQR)	67 (52–76)	67 (50–75)	66 (51–78)	67 (53–76)
**Ethnicity, no. (%)**				
White	864 (71)	122 (74.8)	291 (72.9)	451 (69.7)
Asian	17 (1.4)	1 (0.6)	11 (2.8)	5 (0.8)
Black	86 (7.1)	12 (7.4)	17 (4.3)	57 (8.8)
Hispanic	30 (3.0)	4 (2.5)	14 (3.5)	19 (2.9)
Other	205 (16.9)	24 (14.7)	66 (16.5)	115 (17.8)
SAPSII, median (IQR)	46 (36–57)	44 (29–54)	44 (34–54)	48 (39–59)
SOFA, median (IQR)	6 (4–9)	5 (3–8)	6 (4–8)	7 (5–10)
ECI, median (IQR)	8 (3–14)	8 (3–13)	7 (3–14)	9 (4–14)
GCS, median (IQR)	8 (6–11)	9 (6–11)	8 (6–11)	5 (5–11)

IQR, interquartile range; ECI, Elixhauser Comorbidity Index; CCU, coronary care unit; CSRU, cardiac surgery recovery unit; MICU, medical intensive care unit; TSICU, trauma/surgical intensive care unit; SICU, surgical intensive care unit; SOFA, Sequential Organ Failure Assessment; SAPSII, Simplified Acute Physiology Score; GCS, Glasgow Coma Scale.

### Primary outcome

Patients who underwent tracheostomy between 5 and 10 days had lower 90-day mortality comparing to patients underwent tracheostomy after 11 days. While there was no statistical difference in patients who underwent tracheostomy between 5 and 10 days and patients who underwent tracheostomy within 4 days. Detailed multiple comparison results are shown in Table 2. To analyze factors that might affect 90-day mortality, univariate and multivariate COX proportional hazard regression analyses were used. Factors that might influence mortality were included in the analysis, including age, gender, tracheostomy timing, ICU type on the day of tracheostomy, admission type, GCS score on admission, SOFA score, SAPS-II score and ECI score. Detailed results are shown in Table 3. After adjusting age, SAPSII score and ECI score, tracheostomy timing was still an independent risk factor of death.

**TABLE 2:** Primary outcome of patients at different tracheostomy timing.

	Tracheostomy timing			
	
Outcome	≤4 days	5–10 days	≥11 days	*P*-value
90-days mortality, no. (%)	40 (24.5%)	84 (21.1%)^a^	206 (31.8%)	<0.001

^a^Significantly lower than those underwent tracheostomy after 11 days.

**TABLE 3:** Univariate and multivariate COX regression.

	Univariate analysis		Multivariate analysis	
		
	Unadjusted HR (95% CI)	*P* -value	Adjusted HR (95% CI)	*P* -value
Age		<0.001		<0.001
≤52	Reference			
52–66 (including 66)	2.5 (1.5–4.0)		1.8 (1.1–3.0)	0.016
66–76 (including 76)	4.5 (2.9–7.0)		3.0 (1.8–4.7)	<0.001
>76	6.6 (4.3–10.3)		4.4 (2.8–7.0)	<0.001
ECI score		<0.001		<0.001
≤3	Reference			
3–8 (including 8)	1.6 (1.1–2.3)		1.3 (0.9–1.9)	0.226
8–14 (including 14)	2.2 (1.5–3.1)		1.6 (1.1–2.3)	0.009
>14	3.1 (2.2–4.4)		2.1 (1.5–3.1)	<0.001
Tracheostomy time		0.001		0.004
≤4 days	Reference			
5–10 days	0.9 (0.6–1.3)		0.8 (0.6–1.2)	0.369
≥11 days	1.4 (1.0–2.0)		1.3 (0.9–1.8)	0.181
ICU type on the day of tracheostomy		<0.001		0.08
TSICU/SICU	Reference			
CCU/CSRU	1.9 (1.4–2.5)			
MICU	1.5 (1.2–2.0)			
Admission type		0.063		0.196
Emergency	Reference			
Urgent	1.8 (1.1–2.7)			
Selective	1.2 (0.8–1.7)			
Gender		0.922		/
Female	Reference			
Male	1.0 (0.8–1.2)			
SAPSII		<0.001		0.012
≤36	Reference			
36–46 (including 46)	2.5 (1.6–3.8)		1.5 (1.0–2.3)	0.062
46–57 (including 57)	3.7 (2.5–5.5)		1.9 (1.2–2.9)	0.005
>57	4.4 (3.0–6.6)		1.9 (1.2–3.0)	0.003
SOFA		0.002		0.965
≤4	Reference			
4–6 (including 6)	1.3 (0.9–1.9)			
6–9 (including 9)	1.5 (1.1–2.1)			
>9	1.8 (1.3–2.5)			
GCS in admission		0.814		/
≥12	Reference			
9–11	0.9 (0.7–1.3)			
≤8	0.9 (0.7–1.2)			

HR, hazard ratio; CI, confidence interval; ECI, Elixhauser Comorbidity Index; CCU, coronary care unit; CSRU, cardiac surgery recovery unit; MICU, medical intensive care unit; TSICU, trauma/surgical intensive care unit; SICU, surgical intensive care unit; SOFA, Sequential Organ Failure Assessment; SAPSII, Simplified Acute Physiology Score; GCS, Glasgow Coma Scale.

**FIGURE 3:**
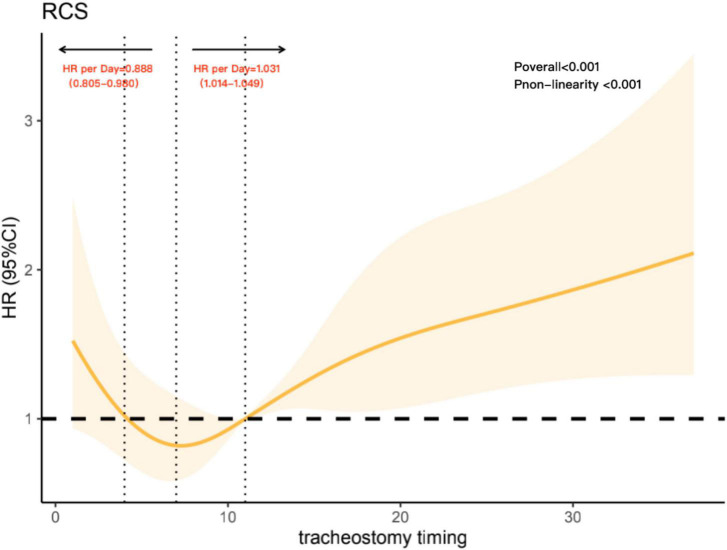
Association with tracheostomy timing and mortality, adjusted variables including age, Simplified Acute Physiology Score (SAPS) II score, and Elixhauser Comorbidity Index (ECI) score. The reference point is the median tracheostomy timing and knots at the 5th, 27.5th, 50th, 72.5th, and 95th centiles of tracheostomy timing. The vertical line on the left shows tracheostomy timing around Day 4, while the vertical line in the middle shows tracheostomy timing around Day 8. The vertical line on the right shows tracheostomy timing on Day 11; it intersects the horizontal dotted line, and the intersection point represents the reference point.

To further analyze the non-linear association between tracheostomy timing and the risk of 90-day all-cause mortality, we constructed restricted cubic spline model. In this model, the association between tracheostomy timing and all-cause 90-day mortality was U-shaped, as shown in Figure 3 (*P*_overall_ < 0.001), adjusted variables including age, SAPSII score, and ECI score. The risk of 90-day all-cause mortality decreased with the extension of tracheostomy timing until approximately 8 days after intubation and then started to increase afterward (*P* for non-linearity < 0.001). Before Day 8, the hazard ratio delayed each day was 0.888 (0.805–0.980), while after Day 8, the hazard ratio delayed each day was 1.031 (1.014–1.049). Meanwhile, around Day 4 to Day 11 after intubation, the hazard ratio was less than 1.

**FIGURE 4:**
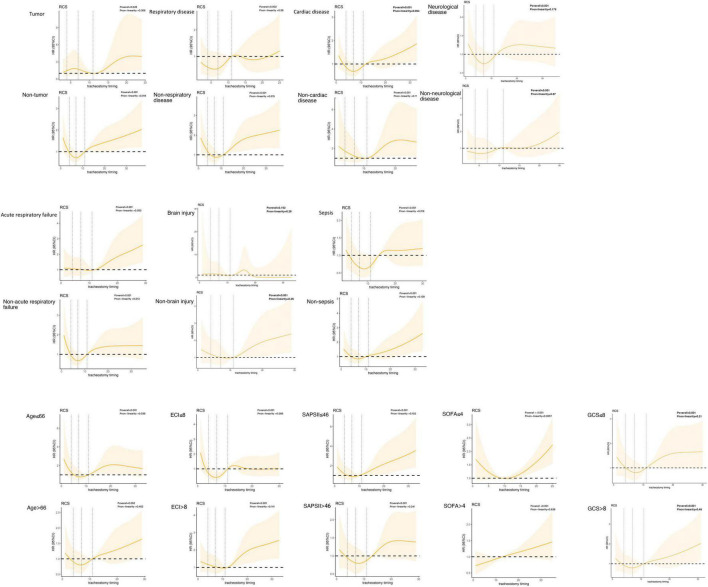
Association with tracheostomy timing and mortality in different patient groups.

Then, we discussed the relationship between the tracheostomy timing and 90-day all-cause mortality for patients with different comorbidities to further refine the possible causes of the U-shaped curve. Comorbidities included tumor or non-tumor, respiratory disease or non-respiratory disease, cardiac disease or non-cardiac disease, neurological disease or non-neurological disease. According to the GCS scores on admission, we analyzed the association between tracheostomy timing and mortality in patients with severe consciousness disorders (GCS ≤ 8) and patients with mild to moderate consciousness conditions (GCS > 8). We also analyzed three particular diseases: sepsis, acute respiratory failure, and brain injury. Patients of different ages, ECI scores, SAPS II scores and SOFA scores were also analyzed. There was a strong linear relationship between SOFA ≥ 4 and 90-day mortality, which declared that in those patients, early tracheostomy might have more benefits. For patients younger than 66, ECI less than 8 and SOFA less than 4, there were a non-linear relationship between tracheostomy timing and 90-day mortality. While in the four kinds of comorbidities, different levels of consciousness and three kinds of diseases, we did not find such a non-linear relationship (Figure 4).

### Secondary outcome

As showed in Table 4, patients who underwent tracheostomy within 4 days had less hospital and ICU LOS, less length of MV, and fewer sedative and analgesia days. Patients who underwent tracheostomy within 5–10 days were more likely to be diagnosed with VAP. Multiple comparisons were also performed and *P*-value after adjustment by Bonferroni’s method were showed in Supplementary Table 1.

**TABLE 4:** Secondary outcome.

	Tracheostomy timing			
	
Outcome	≤4 days	5–10 days	≥11 days	*P*-value
In-ICU mortality, no. (%)	14 (8.6%) a	35 (8.8%) a	108 (16.7%) b	<0.001
30-days mortality, no. (%)	16 (9.8%) a,b	41 (10.3%) b	110 (17%) a	0.003
Hospital LOS, median (IQR)	18.7 (12.8–29.5)	21.7 (15.1–30.2)	32.1 (24.4–43.8)	<0.001
ICU LOS, median (IQR)	14.8 (7.7–24.0)	16.0 (12.1–23.6)	28.2 (21.7–38.0)	<0.001
Length of MV, median (IQR)	8.4 (4.2–15.1)	11.5 (8.6–16.8)	22.3 (17.1–30.3)	<0.001
MV free days, median (IQR)	4.6 (2.2–8.8)	4.1 (1.8–7.1)	4.2 (1.8–8.5)	0.414
Sedative days, median (IQR)	5.0 (3.0–9.0)	7.0 (4.0–10.0)	11.0 (6.0–17.0)	<0.001
Analgesia days, median (IQR)	5.0 (3.0–10.0)	9.0 (5.0–13.0)	12.0 (6.0–21.0)	<0.001
Sedation and analgesia free days, median (IQR)	6.9 (2.1–11.6)	7.0 (3.5–14.3)	13.5 (6.4–22.9)	<0.001
VAP rate, n (%)	15 (9.2) a,b	49 (12.3) b	44 (6.8) a	<0.001

IQR, interquartile range; MV, mechanical ventilation; LOS, length of stay; VAP, ventilator associated pulmonary. Corner markers a and b represent the results of multiple comparison. In each outcome, the same corner markers mean no statistical difference between the two groups, while different corner markers mean statistical difference between the two groups.

## Discussion

In this retrospective cohort study, we sought to explore the association between tracheotomy timing and mortality. We retrospectively analyzed MV patients with tracheostomy records in MIMIC-III database and found that compared to patients who underwent tracheostomy within 4 days and after 11 days, patients who underwent tracheostomy within 5–10 days had less 90-day mortality. Meanwhile, the association between tracheostomy timing and all-cause 90-day mortality was U-shaped, reaching the lowest risk around Day 8 and then increased thereafter.

In published clinical studies, researchers defined tracheostomy timing differently. Some RCTs limited early tracheostomy to within 5 days of MV and late tracheostomy to 10 days after MV (30, 31). Some studies also used a single time point as the time limit for distinguishing early and late tracheostomy (6, 8, 32, 33). However, regardless of the classification, the trajectory of change between continuous changes in tracheotomy timing and mortality risk in MV patients was ignored. Artificially segmenting the timing of tracheotomy may not only result in loss of information but also lead to low statistical efficiency, resulting in bias of results (34, 35). Additionally, guidelines have suggested that tracheostomy in intensive care should not be performed before the fourth day of MV, since early tracheostomy before the fourth day of mechanical ventilation is not associated with decreases in mortality (36). Therefore, we believe that there may be a non-linear relationship between tracheostomy timing and the risk of death. In this study, we used restricted cubic spline analysis to explore this relationship. After adjusting other influencing factors, the non-linear dose-response relationship was presented objectively and cleanly (37). We chose Day 11 as the reference point for restricted cubic spline analysis, not only because Day 11 is the median tracheostomy timing, conforming with the analysis of restrictive cubic spline routine (29) but also because previous studies used Day 10 to distinguish tracheostomy timing and obtained a positive result (6, 8), suggesting that approximately Day 10 may be the boundary between the risk of death. In this research, results suggest that performing tracheostomy between day 4 and 11 after intubation may be a safer choice and that proceeding with tracheostomy too early or too late would significantly increase the risk of 90-day all-cause mortality.

We also tended to find the potential reason for the U-shaped curve by dividing patients into different disease groups and different severity groups. There was a strong linear relationship between SOFA ≥ 4 and 90-day mortality, while in patients with lower SOFA scores, the relationship was still non-linear. Which suggested in patients with less severe disease, tracheostomy should be delayed, while in severe patients, tracheostomy should be performed as soon as possible. Considering the influence of consciousness level on tracheostomy strategy, we also compared the differences in correlation between tracheostomy and mortality in patients with different consciousness levels according to GCS scores. However, no significant non-linear relationship was found in patients with different GCS scores. And in the four comorbidities and three diseases we focused on, no significant non-linear relationship was found either. To explore the non-linear relationship between the tracheostomy timing and mortality in different diseases, more studies are needed.

In our study, it is important to note that after Day 11, which is the median tracheostomy timing, the risk of mortality increased nearly linearly, thus supporting the idea that late tracheostomy may increase the risk of mortality in MV patients. For early tracheostomy, based on the U-shaped curve between the tracheostomy timing and 90-day all-cause mortality, studies focusing on the influence of early tracheostomy and ultra-early tracheostomy on mortality could be regarded as future research directions.

This study found that early tracheostomy could significantly shorten LOS in the ICU and hospital, shorten MV duration, and reduce the need for sedation and analgesia, which was consistent with previous research results (38–40). Although the free days of sedation and analgesia in the late group were longer than the free days of sedation and analgesia in the early group, considering the huge gap between the LOS of these two groups in the ICU, the result is explainable. In our study, patients who underwent tracheostomy within 5–10 days suffered a higher rate of VAP than late patients, which is inconsistent with previous research results (15) and clinical cognition. In this study, the diagnosis of VAP was based on the diagnosis clearly recorded in the ICD-9 code in the database. Unfortunately, since the time of diagnosis was not recorded in the MIMIC-III database, we were unable to determine the time relationship between the occurrence of VAP and tracheostomy, and we were also unable to determine whether the cause of tracheostomy was due to the occurrence of VAP.

This study provides a large sample size, real-world clinical study that focuses on factors influencing the outcome of different tracheostomy timings. To the best of our knowledge, this is the first study introducing the combination of restricted cubic splines and Cox regression into exploring the relationship between tracheostomy timing and mortality. We not only discussed the relationship between tracheostomy timing and mortality but also analyzed the trend between tracheostomy timing and mortality risk and focused on the non-linear relationship between tracheostomy timing and mortality risk. Meanwhile, previous studies excluded patients who failed extubation several times prior to tracheostomy, which may have caused selection bias artificially. This study makes up for the defects of this type of research. In addition, according to the relationship between intubation, extubation and tracheostomy time, a new definition of tracheostomy timing was adopted. Patients with repeated endotracheal intubation before tracheostomy were also included in the study, which was more in line with clinical practice. However, as a single-center retrospective study, this study has limitations. First, the database we used was the MIMIC-III database. Although there have been many high-quality studies based on this open-source database (41, 42), the source of the MIMIC-III database was only one hospital. The timing of tracheostomy depends on the experience of the physician and the standardized procedures of the hospital. Therefore, the applicability of applying the results of this study in other situations will be reduced. Second, we mainly focused on the relationship between tracheostomy timing and mortality in tracheostomized patients. Patients who died before tracheostomy were not included in this research. Therefore, we cannot determine whether the absence of tracheostomy was one of the risk factors for death in such patients, which may cause particular bias. Third, as a retrospective study, we explored the correlation between tracheostomy timing and mortality, but the causal relationship between the two needs to be confirmed by further RCTs.

## Conclusion

The tracheotomy timing showed a U-shaped relationship with all-cause mortality, and the risk of mortality was lowest on Day 8, but a causal relationship has not been demonstrated.

## Data availability statement

Publicly available datasets were analyzed in this study. This data can be found here: Johnson A., Pollard T., and Mark R. (2016). MIMIC-III Clinical Database (version 1.4). PhysioNet (https://doi.org/10.13026/C2XW26).

## Ethics statement

The establishment of this database was approved by the Massachusetts Institute of Technology (Cambridge, MA) and Beth Israel Deaconess Medical Center (Boston, MA), and consent was obtained for the original data collection. Therefore, the ethical approval statement and the need for informed consent were waived for this manuscript by the Institutional Review Board of Beijing Tiantan hospital. The study was designed and conducted in accordance with relevant guidelines and regulations (Declaration of Helsinki).

## Author contributions

J-XZ and G-QC conceptualized the research aims and guided the literature review. J-RC and H-RG improved the design and extracted data from the MIMIC-III database. Y-MZ verified the data. Y-LY and YW participated in processing the data and doing the statistical analysis. J-RC wrote the first draft of the manuscript. LZ and J-XZ provided comments and approved the final manuscript. All authors read and approved the final manuscript.

## Footnotes

1
http://www.Rproject.org
