# Supplementary material for: A U-shaped association of tracheostomy timing with all-cause mortality in mechanically ventilated patients admitted to the intensive care unit: A retrospective cohort study
Source: Front Med (Lausanne). 2022 Dec 14;9:1068569. doi: 10.3389/fmed.2022.1068569 (PMC9794610; doi:10.3389/fmed.2022.1068569)
Supplement: Supplementary file 1 [file Table_1.DOCX]

Supplementary Material

# Supplementary Data

**Supplementary Table 1**. P value in multiple comparison adjusted by Bonferroni’s method

| Adjusted P value | | | |
| --- | --- | --- | --- |
| Outcome | ≤4days VS 5-10days | 5-10 days VS ≥11days | ≤4days VS≥11days |
| Hospital LOS | 0.024 | <0.001 | <0.001 |
| ICU LOS  Length of MV  Sedative days  Analgesia days  Sedation and analgesia free days | 0.009  <0.001  0.003  0.459  1 | <0.001  <0.001  <0.001  <0.001  <0.001 | <0.001  <0.001  <0.001  <0.001  <0.001 |
